# Supplementary material for: Public perceptions and expectations: Disentangling the hope and hype of organoid research
Source: Stem Cell Reports. 2023 Mar 30;18(4):841–52. doi: 10.1016/j.stemcr.2023.03.003 (PMC10147824; doi:10.1016/j.stemcr.2023.03.003)
Supplement: Document S1. Tables S1–S4 [file mmc1.pdf]

Stem Cell Reports, Volume *18*

Supplemental Information

Public perceptions and expectations: Disentangling the hope and hype of organoid research

Tine Ravn, Mads P. Sørensen, Emma Capulli, Panagiotis Kavouras, Renzo Pegoraro, Mario Picozzi, Louise I. Saugstrup, Eleni Spyrakou, and Vana Stavridi

SUPPLEMENTAL EXPERIMENTAL PROCEDURES

Design: Format and setting

The objective of conducting deliberative workshops was to explore and elicit different views on the use and derivation of organoids and organoid research to understand the public opinion (i.e., worries, concerns, fears, uncertainty, and expectations) and key ethical issues and implications related to organoids from the point of view of citizens, vulnerable groups, patients, donors, and CSOs. As a participatory method, deliberative workshops facilitate informed deliberations and a (co) production of opinions based on expert information and in-depth dialogue (for more details, please see separate research protocol, Ravn and Sørensen, 2021). The deliberative workshop was designed to explore the following research questions:

- ***How do non-professional stakeholders and the lay public perceive organoids and organoid research?***
  - What are the participants' main worries, fears, and expectations concerning organoid research?
  - How do participants conceptualize and understand organoids (i.e., persons vs. things, moral status, mythological aspects)?
  - What are the perceived current and future benefits of organoid research according to the participants?
  - Which kinds of ethical issues or research pose concern for the participants? (i.e., particular organoid types and uses; particular ethical issues such as informed consent, ownership, and commercialization)?

The three workshops were structured as a two-phase deliberation, each phase including small group and plenary discussions (please see overview, Table S1).

Table S.1. Structure and content of the deliberative workshops. Experimental procedures.

**Phases**	**Deliberative elements**	**Specifications for small-group deliberations**
**Phase 1: Attitudes and conceptualizations**	• Introduction (ethical issues)<br>• Short ice-breaker exercise<br>• Short questionnaire<br>• Presentation by experts<br>• Small-group deliberations<br><br>• Q&A session with experts<br>• Small-group deliberation<br>• Rapporteurs from each group relay a summary of the group discussion<br>• Short summation of the first part of the deliberation	Collective group preparation of three questions to experts<br><br>Deliberation based on two guiding questions regarding attitudes toward and conceptualization of organoids:<br><br>1) What do you think of organoids? What are organoids? How can we best describe them? Do you think of them as human, as a thing, or as something in-between?<br>2) Discuss the feelings organoids evoke in you. Which words come to mind when you think about organoids? Are organoids something to be feared – or do you see a hope in them? Which worries and/or positive expectations do you have regarding organoids?

**Phase 2: Ethical implications and recommendations**	• Introduction to the second part of the deliberation<br>• Individual reading of dilemmas and individual reflections<br>• Small-group deliberations<br>• Rapporteurs from each group relay a summary of the group discussion<br>• Short presentation of recommendation posters<br>• Plenum deliberation<br>• Short questionnaire<br>• Conclusion and evaluation (debriefing)	Presentations of two dilemmas related to a) Consent, ownership, and compensation and b) Cerebral organoids and the consciousness issue<br><br>A) Discussion of dilemmas based on guiding questions<br>B) Exercise. List of recommendations for important ethical issues, challenges and concerns to be taken into account in regard to organoid research<br>Guiding question:<br>1) What are the most important ethical issues that a regulatory framework for organoid research must address?

The first phase also entailed expert presentations and a Q&A session. Table S2 summarizes the focus of the expert presentations. Professional moderators and national project partners helped facilitate the small-group discussions. Subsequent to these deliberations, a rapporteur from each group relayed a summary to the following plenary discussions for collective deliberation. In the second phase, participants also provided a set of recommendations for important ethical issues, challenges, and concerns to be taken into account in future organoid research (see Table S2).

The three deliberative workshops followed the same protocol and design. All materials were developed by the study coordinators (TR, MPS) and translated and adapted if necessary by research partners locally. Participants were provided with a set of different materials prior to the workshop in order to safeguard consent, promote voluntariness, protect privacy and confidentiality, and inform participants accurately (all documents are available upon request):

- A 23-page information kit was sent out one week prior to the date of the workshop to help facilitate an informed dialogue. The kit included three short texts:
  1. What are organoids? How are they made, and how can they be used now and potentially in the future? The text broadly introduced both techniques of pluripotent stem cells (PSCs) and adult stem cells (AdSCs) for generating organoids. In general, the introduction referred to both and did not discuss detailed overlap or distinct ethical and legal implications except for a discussion on embryoids.
  2. What are some of the ethical implications of organoid research? This section included an overview of ethical issues in relation to animal experiments, different types of organoids (i.e., cerebral organoids and embryoids), biobanks, ownership and informed consent, and research integrity and public communication.
  3. How can we understand organoids conceptually? This overview introduced questions concerning the conceptual uncertainty of organoids as hybrid entities.
  The information material also included suggestions for extra material, including links to two short YouTube videos on organoid research: 1) A 3-minute introduction to organoid research by Lie and Belmonte and the New England Journal of Medicine group and a 4-minute TED-Ed animation of “What are mini-brains?” by Madeline Lancaster.
- Invitation to participate
- Information letter to participants
- Consent form
- A note on how Aarhus University processes personal data
- Detailed workshop agenda and workshop material

Table S.2. Overview of expert presentations. Experimental procedures.			
	**Italy**		
**Experts**	**Affiliation**	**Research area**	**Description of presentation**
Dr. Teresa Rinaldi	Associate professor. Department of Biology and Biotechnology “Charles Darwin” of the Sapienza University of Rome	Research and teaching within pharmacogenomics, fermentation biotechnology, and applied geosciences and bio conservation laboratory.	The presentation entitled “Organoids” introduced the topic in general terms, explaining what organoids are, by which cells, and how they are produced and the various types of organoids that exist today. The expert illustrated the possible applications of this new biotechnology and the ethical questions it raises. It was illustrated how organoids can be used as a model for the study of genetic diseases, infectious diseases, and for the study of tumors. Finally, a number of ethical issues raised by the use of organoids were pointed out, such as the moral and legal status of organoids, the use of gene editing, the creation of chimeras, marketing, and biobank storage.
Dr. Andrea Lavazza	Senior Research Fellow in Neuroethics at the Centro Universitario Internazionale di Arezzo and Adjunct Professor at the University of Pavia. He is also a lecturer at the Master of Forensic Psychopathology and Neuropsychology at the Faculty of Psychology, University of Padua.	Research in philosophy of mind and neuroethics. His main fields of interest are free will, human enhancement (including gene editing), memory-modulation, cognitive liberty, and human cerebral organoids.	The presentation entitled “Cerebral organoids and ethical questions” focused on a particular type of organoid: cerebral organoids. A number of ethical issues were outlined, some relating to organoids in general (e.g., issues relating to informed consent and ownership of the cells used) and some specifically relating to cerebral organoids (e.g., the transfer of cerebral organoids into animal models chimeric). After an overview of the various issues raised by brain organoids, the question of whether organoids can develop a form of consciousness and how this possibility affects their moral status was explored.
	**Greece**		
Dr Olga Tzortzatou-Nanopoulou	Legal counsellor and President of the Intellectual Property Committee of the Biomedical Research Foundation of the Academy of Athens (BRFAA), European Commission Expert	Specialized in GDPR/informed consent issues and IP/patents:	The presentation with the title “Information society and the current role of bioethics: Personal data and informed consent” focused on the existing regulation on biomedical research, regarding informed consent, in particular. The expert referred to the International Chapter of Principles for Sharing Data and Bio-specimen, to the GDPR regulation and crucial bioethical questions. She also presented relevant ELSI issues as well as the role of Bioethics Committees. Finally, some cases involving violations of the

			aforementioned regulations were presented.
Dr Miltos Ladikas	Senior researcher at the Institute of Technology Assessment and Systems Analysis, Karlsruhe Institute of Technology, Germany. Adviser to the European Commission, the European Research Council, the European & Developing Countries Clinical Countries Partnership, and a number of National Research organizations on social-ethical issues in science & technology developments	Specialized in Global aspects of Technology Assessment, Responsible Innovation, Ethics in Science and Technology Policy, as well as, Science Diplomacy	The presentation with the title "Technology assessment and policy making" was relevant to connecting the Technology Assessment with policy making. The expert explained the policy making procedure for a new technology and how the public opinion is introduced to this procedure. He proceeded to the description of the policy making system of some European countries and notified the importance of the public participation to the technology assessment and policy making.
	**Denmark**		
Dr. Thomas Lykke-Møller Sørensen	Associate Professor at the Department of Biological & Chemical Engineering, Aarhus University	Research in molecular biology and biotechnology, organoid technologies, and imagining biotechnology	The presentation was based on lab experiences working with organoids. During the presentation, the participants were presented with information about the development in organoid technology and how organoids are grown and used in laboratories. The expert presented different kinds of organoids (e.g., bowel, brain, and cancer) and gave different examples of how organoids are used. The presentation included pictures and videos of organoids.
Dr. Morten Dige	Associate Professor at the School of Culture and Society, Aarhus University	Research in bioethics and moral philosophy	This presentation focused on different ethical questions related to organoid research. The main focus concerned questions regarding moral status, informed consent related to the use of human tissue, and whether it is problematic to make artificial human organoids in general. The presentation included information on what moral status is, and how attitudes to who/what has moral status have changed over time. The participants were also introduced to different types of consent and how consent relates to governance in organoid research, and the relevance of commercialization, patents, and consent as issues in

			relation to the handling, distribution, and storing of stem cells.

Sampling and recruitment

Based on a separate research protocol on sampling strategy and procedures for recruitment (for details, see Ravn and Sørensen 2021a), national partners recruited participants for the deliberative workshops locally. A common excel sheet was developed to document and facilitate the recruitment process. To secure diversity in representation, a purposeful maximum-variation strategy was applied and the following participant categories were selected and included:

- The general public (e.g. variation as to age, gender, socio-economic group, ethnic background, and religious view. Members of the LGDBQ+ community was also represented)
- Vulnerable groups (e.g. including relatives to patients with genetic diseases, parents to children with genetic diseases, patients self-identifying as vulnerable)
- Patients (including e.g. patients with cystic fibrosis, ALS, gastrointestinal diseases, or cancer). Patients could also be donors.
- Donors (healthy donors donating different types of biological material)
- Civil society organizations (including e.g. religious organizations, patient organizations, science outreach organizations, blood donation organisation, students associations, consumers association)

A broader representation between participants unfamiliar with organoids and key non-professional actors (or enclave groups) representing minor groups of the public with particular experiences and/or interests in organoid research was seen as valuable to elicit a range of attitudes and explore a diverse set of organoid perceptions and understandings. In addition to ensuring variation among types of participant categories, we sought to secure diversity across age, gender, socio-economic groups, ethnic background, and religious views. The geographical coverage in terms of workshop locations was also sought to include cultural and religious variation and difference regarding the role of science in society. Table S3 provides an overview of the socio-demographic characteristics of the 51 participants participating in one of the three deliberations.

Table S3: Socio-demographic characteristics of the 51 participants. Table 1.

**Socio-demographic information on participants – locally and combined**	**Sub-categories**	**Countries**			**Total per sub-category**
		**Denmark**	**Greece**	**Italy**	
Distribution across participant categories	General public	4	9	3	**16**
	Vulnerable group	6	-	2	**8**
	Patient	5	1	5	**11**
	CSO	8	1	9	**18**
	Donor	3	1	1	**5**
	Not answered	-	-	-	**-**
	Total	26	12	20	**58**[1]
Age distribution	18–30	3	2	4	**9**
	31–40	2	6	4	**12**

[1] 51 people participated in the workshops. However, some participants (in Denmark and Italy) checked more than one box to categorize themselves (e.g., both ‘CSO’ and ‘patient’). The 20 Danish participants checked 26 boxes, while the 19 Italian participants checked 20 boxes.

	41–50	3	3	3	**9**
	51–60	5	-	3	**8**
	61–70	4	1	3	**8**
	71–80	3	-	1	**4**
	81+	-	-	-	**-**
	Not answered	-	-	1	**1**
Gender distribution	Male	13	7	12	**32**
	Female	7	4	6	**17**
	Non-binary	-	1	-	**1**
	Not answered	-	-	1	**1**
Religiosity	Not religious	4	7	5	**16**
	Christian, Orthodox	1	3	-	**4**
	Christian, Catholic	1	-	10	**11**
	Christian, Protestant	12	-	-	**12**
	Christian, other	-	-	-	**-**
	Muslim	1	-	1	**2**
	Other	-	-	-	**-**
	Not answered	1	2	3	**6**
Participants' relation to job market	Employed, low income	-	1	2	**3**
	Employed, middle income	5	9	8	**22**
	Employed, high income	4	1	1	**6**
	Part-time employed	2	-	-	**2**
	Unemployed	-	1	-	**1**
	Retired	6	-	3	**9**
	Student	2	-	3	**5**
	Self-employed	1	-	-	**1**
	Not answered	-	-	2	**2**
Ethnicity/ nationality	Danish	18	-	-	**18**
	Greek	-	12	-	**12**
	Italian	-	-	16	**16**
	European (other than Danish, Greek, and Italian)	2	-	1	**3**
	Non-European	-	-	1	**1**
	Not answered	-	-	1	**1**

Participants were recruited using different strategies:

- Representatives from the public were recruited through a diverse set of media outlets, such as Facebook groups, Twitter, LinkedIn, networks, and well as through political organizations, student organizations, minority organizations etc.
- Vulnerable groups, donors and patients were recruited through patient organizations, support networks, donation organizations, flyers in outpatient clinic waiting rooms, and networks of clinicians.
- CSOs were contacted through existing networks, different associations, or directly through organizational gatekeepers.

In the recruitment and engagement of participants, attention was paid to the need for increased sensitivity to individual and contextual circumstances while adhering to the "recruitment etiquette"

building on the Belmont principles (Gyure et al. 2015, p. 2). Workshop locations were chosen to minimize travel for participants. All travel costs and potential accommodation expenses were reimbursed, and the workshops included catering and a post-workshop dinner. Participants were not given an honorarium but did receive a gift bag as a thank you for their participation.

Data Analysis

The deliberative workshops were audio-recorded for subsequent transcription, data coding, and within-case analysis. National deliberations were locally transcribed verbatim, coded, and analyzed according to a common preliminary codebook, analytical strategy, and template. The within-case analyses were then imported to the NVivo 12 software program and thematically coded (by TR) according to the main- and sub-themes conveyed, building on the pre-defined codebook deductively derived from the research questions, workshop themes, and guiding questions applied. The coding strategy also remained open to inductively derived codes emerging through the coding process to allow for new themes and across case explorations. The across-case analysis included a thematic comparison of the three deliberations, identified differences, and similarities in terms of issues raised, as well as explored contextual and conceptual variation.

Pre- and Post-Deliberation Questionnaire

As part of the deliberative workshops, the participants were asked to fill out a questionnaire in the morning, at the beginning of the workshop before the deliberations began, and again after the deliberations, at the end of the day. The questionnaire included four questions regarding the participants' understanding of and feelings toward organoids as well as questions on ownership and consent, which were asked both before and after the deliberations to measure changes in opinions as a result of the workshop deliberations (see Table S4 for results). The first part of the questionnaire also included socio-demographic questions (see results in Table S3).

Questionnaire to participants in deliberative workshops on organoids

QUESTIONS FOR THE MORNING

1. WHICH PARTICIPANT CATEGORY BEST DESCRIBES YOU?

a. VULNERABLE GROUPS (E.G., PARENTS TO CHILDREN WITH GENETIC DISEASES)
b. PATIENT (E.G., PATIENTS WITH GENETIC DISEASES SUCH AS CYSTIC FIBROSIS, CANCER, NEUROLOGIC DISEASES, GASTROINTESTINAL DISEASE, MACULAR GENERATION AMONG OTHERS)
c. DONORS (HEALTHY DONORS DONATING DIFFERENT TYPES OF BIOLOGICAL MATERIAL)
d. CIVIL SOCIETY ORGANIZATIONS, INCLUDING RELIGIOUS ORGANIZATIONS
e. GENERAL PUBLIC
f. PREFER NOT TO ANSWER THIS QUESTION

2. WHICH AGE GROUP DO YOU BELONG TO?

a. 18–30 YEARS OLD
b. 31–40 YEARS OLD
c. 41–50 YEARS OLD

d. 51–60 YEARS OLD
e. 61–70 YEARS OLD
f. 71–80 YEARS OLD
g. 81+ YEARS OLD
h. PREFER NOT TO ANSWER THIS QUESTION

3. **WHICH GENDER CATEGORY BEST DESCRIBES YOU?**

a. FEMALE
b. MALE
c. NON-BINARY
d. PREFER NOT TO ANSWER THIS QUESTION

4. **DO YOU CONSIDER YOURSELF RELIGIOUS?**

a. NO
b. YES, CHRISTIAN (ORTHODOX)
c. YES, CHRISTIAN (CATHOLIC)
d. YES, CHRISTIAN (PROTESTANT)
e. YES, CHRISTIAN (OTHER)
f. YES, MUSLIM (ALL TYPES)
g. YES, OTHER (THAN MUSLIM OR CHRISTIAN)
h. PREFER NOT TO ANSWER THIS QUESTION

5. **WHICH LABOR MARKET CATEGORY BEST DESCRIBES YOU?**

a. EMPLOYEE, LOW INCOME
b. EMPLOYEE, MIDDLE INCOME
c. EMPLOYEE, HIGH INCOME
d. EMPLOYEE, PART-TIME
e. UNEMPLOYED
f. RETIRED
g. STUDENT
h. PREFER NOT TO ANSWER THIS QUESTION

6. **WHAT IS YOUR ETHNIC/NATIONAL BACKGROUND?**

a. DANISH
b. GREEK
c. ITALIAN
d. EUROPEAN (OTHER THAN DANISH/GREEK/ITALIAN)
e. NON-EUROPEAN
f. PREFER NOT TO ANSWER THIS QUESTION

7. **WHICH WORDS BEST DESCRIBE AN ORGANOID? PLEASE CHOOSE UP TO THREE ANSWERS**

a. THING
b. HUMAN
c. HALF HUMAN, HALF THING
d. LIVING ORGANISM
e. SOMETHING MECHANICAL
f. ARTIFICIAL
g. NATURAL
h. CLINICAL TOOL
i. RESEARCH TOOL

j. Cell culture
k. Mini-organ
l. Science fiction
m. Frankenstein-like
n. In doubt/don't know
o. Prefer not to answer this question

8. Which of the following words (or word combinations) best describe how you feel about organoids? Please choose two words

a. Fear
b. Hope
c. Positive expectation
d. Worries
e. Progress
f. Danger
g. Excitement
h. Other feelings
i. Does not evoke any feelings
j. Prefer not to answer this question

9. Who owns the organoids?

a. The donor of the cells
b. The researcher/doctor
c. The hospital/research institution/company/biobank
d. The state
e. Don't know
f. Prefer not to answer this question

10. Which type of consent should donors give for the use of their cells for organoid research?

a. Blanket consent (i.e., material is donated without any restrictions)
b. Dynamic consent (i.e., on-going engagement and communication between donors and users)
c. Don't know
d. Prefer not to answer this question

QUESTIONS FOR THE AFTERNOON

11. Which words best describe an organoid? Please choose up to three answers

a. Thing
b. Human
c. Half human, half thing
d. Living organism
e. Something mechanical
f. Artificial
g. Natural
h. Clinical tool
i. Research tool

j. CELL CULTURE
k. MINI-ORGAN
l. SCIENCE FICTION
m. FRANKENSTEIN-LIKE
n. IN DOUBT/DON'T KNOW
o. PREFER NOT TO ANSWER THIS QUESTION

12. WHICH OF THE FOLLOWING WORDS (OR WORD COMBINATIONS) BEST DESCRIBE HOW YOU FEEL ABOUT ORGANOIDS? PLEASE CHOOSE TWO WORDS

a. FEAR
b. HOPE
c. POSITIVE EXPECTATION
d. WORRIES
e. PROGRESS
f. DANGER
g. EXCITEMENT
h. OTHER FEELINGS
i. DOES NOT EVOKE ANY FEELINGS
j. PREFER NOT TO ANSWER THIS QUESTION

13. WHO OWNS THE ORGANOIDS?

a. THE DONOR OF THE CELLS
b. THE RESEARCHER/DOCTOR
c. THE HOSPITAL/RESEARCH INSTITUTION/COMPANY/BIOBANK
d. THE STATE
e. DON'T KNOW
f. PREFER NOT TO ANSWER THIS QUESTION

14. WHICH TYPE OF CONSENT SHOULD DONORS GIVE FOR THE USE OF THEIR CELLS FOR ORGANOID RESEARCH?

a. BLANKET CONSENT (I.E., MATERIAL IS DONATED WITHOUT ANY RESTRICTIONS)
b. DYNAMIC CONSENT (I.E., ONGOING ENGAGEMENT AND COMMUNICATION BETWEEN DONORS AND USERS)
c. DON'T KNOW
d. PREFER NOT TO ANSWER THIS QUESTION

Pre- and Post-Deliberation Questionnaire Results

Table S4 displays the results of the four repeated questions in the Pre- and Post-deliberation questionnaire on participants' understanding of and feelings toward organoids, ownership, and consent.

Table S4. Pre- and post-questionnaire results. Results.

		Denmark		Greece		Italy		Combined	
		Before	After	Before	After	Before	After	Before	After
**Words describing**	**Thing**	-	1	-	-	2	1	2	2
	**Human/person**	1	1	1	-	1	-	3	1
	**Half person/half thing**	-	-	-	-	3	1	3	1

	Living organism	10	14	3	1	7	4	20	19
	Something mechanical	-	-	1	-	0	1	1	1
	Artificial	3	3	6	4	6	1	15	8
	Natural	2	3	1	2	2	2	5	7
	Clinical tool	1	2	2	3	2	4	5	9
	Research tool	7	11	3	7	12	15	22	33
	Cell culture	11	12	7	3	7	6	25	21
	Mini-organ	10	11	1	3	5	3	16	17
	Science-fiction	-	-	-	-	-	-	-	-
	Frankenstein-like	-	-	-	-	-	-	-	-
	In doubt/don't know	3	-	2	1	-	-	5	1
	Not answered	-	-	-	-	1	-	1	-
	**Total**	**48**	**58**	**27**	**24**	**48**	**38**	**123**	**120**
**Feelings related to organoids**	Fear	1	3	2	-	-	-	3	3
	Hope	8	10	5	4	12	14	25	28
	Positive expectations	13	13	7	10	8	9	28	32
	Worries	4	9	3	4	1	1	8	14
	Progress	9	8	3	1	9	12	21	21
	Danger	0	1	1	1	1	1	2	3
	Excitement	5	6	1	-	1	1	7	7
	Other feelings	1	-	-	-	-	-	1	-
	No feelings	-	-	1	1	-	-	1	1
	Not answered	-	-	-	-	2	-	2	-
	**Total**	**41**	**50**	**23**	**21**	**34**	**38**	**98**	**109**
**Who owns organoids?**	The cell donor	5	6	4	-	4	-	13	6
	Researcher/doctor	1	3	1	2	2	1	4	6
	Hospital/research institution/biobank	6	10	1	5	11	16	18	31
	The state	-	4	-	-	1	1	1	5
	Don’t know	11	1	5	3	2	1	18	5
	Not answered	-	-	1	2	2	-	3	2
**Opinions on consent**	Without restrictions	3	3	-	2	3	2	6	7
	Some/certain restrictions	2	4	4	4	4	5	10	13
	Specified consent for certain diseases or areas	4	2	-	1	4	5	8	8
	Ongoing consent	8	10	6	3	6	7	20	20
	Don’t know	3	-	2	-	-	-	5	-
	Not answered	-	1	-	2	2	-	2	3

Supplemental References

Gyure, M.E., Quillin, J.M., Rodríguez, V.M., Markowitz, M.S., Corona, R., Borzelleca, J., Jr, Bowen, D.J., Krist, A.H., and Bodurtha, J.N. (2014). Practical considerations for implementing research recruitment etiquette. *IRB*, *36*(6), 7–12.

Ravn, T., and Sørensen, M.P. (2021). D.4.1. Protocol. Organoid-based research: Engagement, co-creation and validation, HYBRIDA. EU Commission.

Ravn, T., and Sørensen, M.P. (2021a). D.4.2. Report on participant selection and procedures and criteria for recruitment. HYBRIDA. EU Commission.
