# Supplementary material for: Public perceptions and expectations: Disentangling the hope and hype of organoid research
Source: Stem Cell Reports. 2023 Mar 30;18(4):841–52. doi: 10.1016/j.stemcr.2023.03.003 (PMC10147824; doi:10.1016/j.stemcr.2023.03.003)
Supplement: Document S2. Article plus supplemental information [file mmc2.pdf]

Stem Cell Reports
Article
OPEN ACCESS

Public perceptions and expectations: Disentangling the hope and hype of organoid research

Tine Ravn,[1,*] Mads P. Sørensen,[1] Emma Capulli,[2] Panagiotis Kavouras,[3] Renzo Pegoraro,[4] Mario Picozzi,[5] Louise I. Saugstrup,[1] Eleni Spyrakou,[3] and Vana Stavridi[3]
[1]Political Science, Danish Centre for Studies in Research and Research Policy, Aarhus University, Aarhus C, Denmark
[2]Department of Medicine and Surgery, Insubria University, Varese, Italy
[3]School of Chemical Engineering, National Technical University of Athens, Athens, Greece
[4]Pontifical Academy for Life, Rome, Italy
[5]Center for Clinical Ethics, Biotechnology and Life Sciences Department, Insubria University, Varese, Italy
*Correspondence: tr@ps.au.dk
https://doi.org/10.1016/j.stemcr.2023.03.003

SUMMARY

Organoid technologies are rapidly advancing and hold great potential and hope for disease modeling and clinical translational research. Still, they raise a number of complex, ethical questions regarding their current and future use. Patient and public involvement is important in building public trust and helping to secure responsible conduct and valued innovations; nevertheless, research into patient and public perspectives on organoid technologies remains scarce. We report on a first public dialogue on organoid technologies through three cross-country deliberative workshops with a diverse group of stakeholders to identify their perceptions and concerns. Participants generally support organoid technologies on the condition that responsible governance, ethical oversight, and sound informed consent procedures are in place. Yet, a broad set of potential concerns are identified, primarily concerning commercialization, healthcare access, and cerebral organoids. Participants' insights and recommendations can help inform researchers and ethics and policy bodies toward supporting responsible and ethical organoid approaches.

INTRODUCTION

Organoid technologies is a quickly emerging field of research, and while this field is still in its early stages of research, production, and pre-clinical use, it is rapidly advancing and progressing into clinical trial phases with great potential and hope for disease modeling and clinical translational research, such as precision medicine and transplantation. Human organoids also provide unique opportunities for studying both the normal and pathological functioning of organs and human development as a potentially more robust, accessible, and accurate alternative to the application of animal models (Kim et al., 2020; Mollaki, 2021). Still, the derivation and use of organoids raise a number of complex, novel, and familiar ethical questions regarding their current use and potential future applications.

Organoids can be defined as "a three-dimensional structure derived from (pluripotent) stem cells, progenitor, and/or differentiated cells that self-organize through cell-cell and cell-matrix interactions to recapitulate aspects of the native tissue architecture and function *in vitro*" (Marsee et al., 2021, p. 817). As regards future uses of organoids, further advances are expected to lead to more effective medication for specific patient groups, drug discovery, better understandings of disease predisposition through gene variation, testing of experimental gene therapy techniques, and, at some point, possibly also the use of organoids for transplantation (Huch et al., 2017; Kim et al., 2020).

Organoid technologies raise a number of uncertainties and important policy, research integrity, and ethical issues. Organoids can be considered as being hybrid in nature and complex entities, which are not easily categorized within the traditional person/subject and thing/object dichotomies. Being derived from human biological material does not provide them with a status as a person per se. Nonetheless, in a legal and moral aspect it can be argued that they possess not only "instrumental moral value as things, but also relational moral value as persons," questioning also the attribution of moral status to some types of organoids, e.g., embryonic models and cerebral organoids (HYBRIDA Consortium, 2020, p. 11; Gaillard et al., 2021). The transgression of the person/thing dualism prompts conceptual, methodological, and regulatory uncertainty in terms of understanding, evaluating, and regulating organoid entities (HYBRIDA Consortium, 2020). Hence, organoids cannot be regarded as "a morally neutral alternative" (Bredenoord et al., 2017, p. 1) as they are derived from human tissues and cells and raise an array of questions concerning their moral and legal status, particularly for cerebral organoids, assembloids, and embryonic models and questions concerning appropriate types of informed donor consent models. Concerns have also been raised in regard to biobank storage and governance, including issues on data protection, ownership, and commercialization (Baertschi et al., 2020; Bollinger et al., 2021; Bredenoord et al., 2017; Chneiweiss et al., 2022; Mollaki, 2021).

This is an open access article under the CC BY license (http://creativecommons.org/licenses/by/4.0/).

Table 1. Characteristics of participants (n = 51) in workshops

	Italy	Greece	Denmark	Total
Participant category				
Public	5	9	6	20
Patient	3	1	4	8
Donor	3	1	2	6
CSO	5	1	6	12
Vulnerable	3	–	2	5
Total	19	12	20	51
Age (years)				
18–40	7	8	5	20
40–60	7	3	8	18
61+	5	1	7	13
Total	19	12	20	51
Gender				
Female	7	4	7	18
Male	12	7	13	32
Non-binary	–	1	–	1
Total	19	12	20	51

An unexplored topic for organoid technologies concerns the communication with and involvement of a broad range of stakeholders, including publics, patients, civil society organizations (CSOs), industry, and the media. Sound public engagement procedures for the advancement of "sensitive new biotechnologies" have been advised (Caulfield et al., 2016; The National Academies of Sciences, Engineering, and Medicine, 2021, p. 91). For organoid technologies in particular, broad science and society dialogues and interactions are correspondingly called for to help secure responsible conduct and valued innovations within the field and to promote long-term public acceptance of organoid research (Bredenoord et al., 2017; Farahany et al., 2018; Klingler et al., 2022; Lensink et al., 2020). Stakeholder involvement and transparent dissemination of science are key elements in building and substantiating public trust, reducing unjustified worries and hyperbole, and improving policy decision-making processes. Similarly, the reduction of the gaps between realistic and hyped scenarios by aligning understandings of and expectations regarding organoids remains important (Bollinger et al., 2021; HYBRIDA Consortium, 2020; Klingler et al., 2022).

To date, three recent interview studies have been published (two from the Netherlands, one from the US) that enquire into patient perspectives on organoid research (Boers et al., 2018; Bollinger et al., 2021; Haselager et al., 2020). The study by Boers et al. (2018) included 23 interviews with cystic fibrosis (CF) patients examining their experiences and attitudes toward organoid technologies. In the study by Haselager et al. (2020), 28 interviews with patients with neurological diseases or psychiatric disorders and laypersons were conducted on perspectives on cerebral organoids. In Bollinger et al. (2021), 60 interviews with adult patients or parents to patients from different disease populations were conducted.

To facilitate the public dialogue on organoid technologies with a broader and more diverse group of stakeholders, including publics, donors, and vulnerable groups, we conducted three deliberative workshops in three different European countries (Italy, Greece, and Denmark). The key objective was to explore and gain insights into public perceptions of organoids and organoid research (i.e., worries, concerns, fears, uncertainties, and expectations) as well as to identify key ethical issues and implications of organoid technologies. The study also forms part of the European HYBRIDA project (HYBRIDA, 2023) and public perceptions were elicited to help inform the production of an ethical and regulatory framework for the field.

Deliberative workshops are designed to facilitate informed deliberations on a given topic, often of a complex and/or controversial nature, and through evidence assessments and the in-depth examination of issue positions to elicit convergent and divergent perceptions on the particular topic while also supporting understandings of how attitudes develop and possibly change due to expert input and careful deliberations (O'Brien et al., 2020; Steel et al., 2020; The Danish Board of Technology, 2014).

RESULTS

This section reports on the results from the three cross-country deliberations on public perceptions of organoid research conducted in Italy, Greece, and Denmark representing, respectively, different religious, socio-cultural, and science/society contexts (results from the workshops have been reported to the EU Commission). In total, 51 participants partook in the workshops, including citizens, vulnerable groups (examples include parents to children with genetic diseases, relatives to patients with genetic diseases, patients self-identifying as vulnerable), patients, donors, and CSOs (examples include religious organizations, patient organizations, science outreach organizations, student associations, and a blood donation organization). Table 1 provides an overview of selected and distributed socio-demographic information of the participants (for more socio-demographic information on participants, see Table S3 in the supplemental information).

The main objective of the workshop was to explore lay public and stakeholder perceptions of organoid research, including worries, concerns, uncertainties, and expectations; their understandings and conceptualizations of organoids; and key ethical issues and implications in relation to their derivation and use (cf. supplemental information for a detailed methods description). Three broad and interrelated themes are addressed and reported through a thematic analysis: (1) participant attitudes toward organoid technologies vis-a-vis issues of particular ethical concerns, including issues regarding commercialization, governance, and cerebral organoids, (2) how participants conceptualize organoids concerning typology and moral status, and (3) participant recommendations for ethical issues to be taken into account in future guidelines for organoid research. The key findings are substantiated with representative quotations (indicating participant number, category, country, and gender) and relevant results from a pre- and post-deliberation questionnaire distributed in each workshop, carried out to complement and substantiate the perception data and survey deliberative effects (i.e., changes in organoid perceptions and comprehensions). The section focuses on main and convergent views on organoid research, as no discernible differences in stakeholder groups or socio-demographics could be inferred to particular positions on or attitudes regarding organoids. The analysis highlights the few exceptions from this and key differences among the three national contexts.

Perceived benefits of organoid technology

Despite a number of concerns and worries, participants supported the research, production, and use of organoids in general, irrespective of demographic characteristics, religious affiliation, or group representation (i.e., public, patient, donor, vulnerable group, CSO). Participants found the prospects of advancing the existing and future benefits of organoid research promising. They also attached hope and positive expectations to the application of organoids and their contribution to biomedical research.

Participants explicitly mentioned potential benefits pertaining to the development of new and/or advanced treatments of genetic diseases, such as CF and cancer, personalized medicine through patient-derived organoids, preventive medicine and care, the “auto-transplantation” of patient cells, the reduced use of animals in research experiments and drug testing/screening, and increased life expectancy.

The dynamic nature of organoid research

On an aggregate level, participants across the deliberations stressed the dynamic nature of organoid research and, consequently, the continuous need for regular updates and examinations of ethical guidelines, procedures, and frameworks. Cognizant of its state of flux, participants emphasized the exigence for policy, medical, and regulatory procedures to be in alignment with the state of the art of organoid research from the derivation and production of organoids until pre-clinical as well as clinical and translational research. Akin to this key point, a similar call for ongoing societal debate was highlighted as important for public awareness. The same was evident for the involvement and building of continued public trust as organoid technologies progress. Throughout the deliberations, the difference between the current state of organoid research and possible future developments was constantly present in the argumentation and expressed opinions of the participants. Time was viewed as an important factor, and while participants acknowledged that we are in the early phases of organoid research, where organoids are mainly applied as a “model system” (Kim et al., 2020, p. 579) in basic research to study human development, disease modeling, and drug development, they distinguished between current and prospective scenarios. The uncertainty pertaining to the open-ended nature of organoid technology, added to both the expectations and concerns expressed in the deliberations. This finding is similar to the study by Boers et al. (2018, p. 410), in which the “open-endedness of organoid technology,” was also found to characterize the hopes and concerns among CF patients.

Attitudes toward organoid technology and identified ethical implications

Commercialization and healthcare inequalities

A recurring theme during the deliberations was the participants’ unease regarding the excessive commercialization of organoids. In chorus, they expressed concerns as to the exploration of research findings, the nature and management of the economic agendas involved, and as to the beneficiaries of the research. The unease with commercialization should not be perceived as a blanket dismissal of market forces or profit-driven private companies, as several also explicitly mentioned potential positive impacts from the private sector; for instance, in terms of medical advances and drug development. Nonetheless, a key bioethical aspect for participants was the need for proper and responsible governance structures and oversight for both the public and private sectors, although the latter displayed greater caution. The concern for industry monopolization of organoid technologies was tightly interlinked with the concern for increased healthcare inequalities, and comparisons were made with the global case of unequal distribution of COVID-19 vaccines. Two particular apprehensions were voiced in this regard: first, that advances within organoid research might reinforce existing hierarchies among different types of illnesses as to the allocation of funding, research, and treatment

resources; and, second, that therapeutics will be overpriced and not properly distributed within and among populations. Consequently, participants called for organoid research (e.g., knowledge, technologies, treatments) to be broadly accessible, and for promoting health equality.

Privacy issues and data management

In addition to fearing excessive commercialization, participants expressed considerable concern for issues related to breaches of the privacy of personal data, and they envisioned several areas for potential misuse. For such concerns related to private and genetic data protection, the discussions in all three deliberations resided within the context of data storage and donation. Providing a case in point, some participants stated the risk of insurance companies utilizing compromised data to increase insurance premiums or deny their services to potential customers suffering from certain diseases, increasing healthcare divides, and rendering vulnerable people even more exposed to insecurity.

In the Greek deliberation more prominently than in the other two deliberations, participants expressed the view that there is a lack of proper policies and governance on private data security. This worry did not seem to address science per se; rather, it was linked to the conception that science is subject to manipulation by political interests. In all deliberations, albeit not a dominating concern, some participants also mentioned the potential risk of dual use of organoids; specifically, some participants expressed the fear that organoids could be used for biological warfare, including the development of soldiers with augmented physical abilities.

Ownership and remuneration

The question of who the rightful owners of organoids are demanded careful participant reflection, as many were undecided prior to the deliberations. As reflected in the pre- and post-deliberation questionnaire (cf. Table S4 in supplemental information), a majority of the participants viewed hospitals, research institutions, and biobanks as the rightful owners of the donated biological material. This majority increased significantly during the deliberations from 18 to 31 participants, while reducing the number of "do not know answers" from 18 to 5 (see difference between pre- and post-deliberation answers in Table S4 in the supplemental information).

The discussions yielded different perspectives on whether donors should be compensated for their donation. Still, a majority of participants adopted the position that the donation of cells should be regarded as a donation or gift transferred without financial compensation and viewed as comparable with blood donation. The deliberations displayed a strong interlinkage between consent and ownership/remuneration, as the nature of the latter should be contingent on the type of consent given.

Sound governance and ethical oversight

In addition to the ownership and compensation issues, the participants also discussed the type of actors who should be in charge of responsible governance of the donated material. In general, the issue of sound governance procedures traversed the ethical issues and implications discussed.

No unity was reached on governance regarding responsible actors, although a preference for public sector control and oversight received emphasis, as also shown in the recommendations. Despite inconclusiveness in terms of governance responsibilities, unanimity existed regarding the position that the ethical use of cell donations must be guaranteed through strict governance structures, control, and ethical oversight procedures to ensure the ethically responsible, transparent, and safe storage and use of cells, tissues, or organoids in biobanks. Likewise, and inherent to ethical governance, strict procedures for the protection of sensitive personal data were highlighted as a requirement.

Informed donor consent

In general, the issue of informed consent proved significant for the participants and remained key to other topics, such as ownership, commercialization, and societal trust in scientific research. In general, informed consent is an ethical and legal means to safeguard "individuals' and patients' right to autonomy and self-determination" (Mollaki, 2021, p. 4) and was seen as a key instrument to safeguard the ethical and acceptable derivation and use of organoids. For organoid research in particular, uncertainty may arise as to future risks and the specific organoid purposes (e.g., proven/unproven therapies, basic research, etc.) for which individuals or patients are donating their cells (Baertschi et al., 2020; Mollaki, 2021).

In general terms, consent information should be clear, understandable, and precise, and it should explain purpose and use, ownership, and distribution of profit. Moreover, participants spoke to the issue of finding a proper balance between adequate information without over-informing, and several participants referred to how the physical and emotional state of donors might create barriers to processing and relating to too many details.

Different consent strategies can be implemented in organoid biobanking to obtain and store human biological samples. A majority of the participants opted for some restrictions imposed on consent procedures, and only 7 of the 51 participants decided on a broad consent without limitations. Participants were divided as to whether consent should be broad with some restrictions/tiered for certain areas or dynamic/ongoing and reconfirmed if the donation is used for new purposes and applications. In the Greek workshop, a correlation emerged between participants advocating for dynamic consent and concurrently viewing donors as the rightful donation owners entitled to compensation.

Table 2. Exemplary quotations on cerebral organoids

Subject	Exemplary quotations
Cerebral organoids	*There's a set of questions and reservations that are the same for everything, apply to everything in particular about patents, access, and generally everything we have said so far, but I think the cerebral organoids show a noticeable difference only in the case of one perspective—only if one technology could allow an entire brain to reproduce. And that's exactly where the question comes in on how to define consciousness* (religious CSO, male, 61+ years, Greece)<br>*Will it become similar to something living? Something living as we already know it? You know, people with diseases, people who are lying around and can't do much more than breathe? And will it affect how we see those people and the fact that it might be us lying like that someday? There's something about our fundamental perception of human life that's important to think about in this* (religious CSO, female, 61+ years, Denmark)<br>*I'm thinking that if it can contribute to, for example, stopping a disease or limiting a disease—epilepsy, Parkinson's—then the research is okay. But also that you take it … take the personal things in it, then it becomes … For example, Alzheimer's—if you could change something, how would you get your husband back? Would he be the same or would his personality be different?* (CSO, identifies as patient, female, 41–60 years, Denmark)

Cerebral organoids and ensuing concerns

Participants supported and acknowledged the current and potential benefits of developing cerebral organoids, such as the advancement of treatments for neurological and psychiatric disorders. Nonetheless, cerebral organoids raised specific ethical issues, concerns, and uncertainties as to their moral, ontological, and regulatory status (Table 2). A majority of the participants expressed a need to address cerebral organoids as a distinct type of organoids and adopted a cautious positioning, affirming that ethical and regulatory guidelines must be respected.

A main concern in the deliberations was the open question of whether cerebral organoids would be able to exhibit characteristics akin to human sentience, possess cognitive functions, or develop consciousness. As one might expect, the participants did not reach a firm position on this subject and its moral implications, as the development of these technologies is difficult to predict and the definition and clear measurements of consciousness are lacking (Baertschi et al., 2020, p. 6). In their current state, participants did not think that cerebral organoids can be compared with the moral status of either humans or animals. However, the reference to the moral status of animals was widely applied as an analogy, and several participants pointed out that organoids may well be compared with the moral status of animals if further advanced.

Second, a few participants voiced fears that future treatments may have irreversible and adverse effects regarding personality/identity changes and the manipulation of individuals (e.g., political choices). Third, a minor concern was also raised concerning the risk that cerebral organoids would be able to change and weaken our respect for and the valuing of human beings. Particularly, representatives from the church raised this aspect as a more fundamental issue in the Danish debate.

Public conceptualizations of organoids

Conceptual and ontological uncertainties exist as to how to comprehend the nature of organoids, since, as entities, they are not easily characterized according to the familiar persons/thing dualism (see also introduction). Rather, they can be considered hybrid entities, or in-between persons and things, between nature and artifact, since they are complex entities in nature, derived from human cells, which have the ability to grow into self-organizing and organ-resembling structures but without having the status of a human (Gaillard et al., 2021). Participant understandings of organoids, for instance, in relation to their characteristics, moral status, and potential mythological aspects, were defined as inherent to identifying public perceptions. Conceptualizations were explored in the pre- and post-deliberation questionnaire (cf. Table S4 in supplemental information) in which participants were provided with 13 potential organoid descriptors.

The most popular descriptors among the participants post deliberations were as follows (parentheses indicate the number of participants selecting the specific word): research tools (n = 33), cell cultures (n = 21), living organisms (n = 19), and mini-organs (n = 17). In comparison with a more clarified position on ownership upon the deliberations, deliberative effects (i.e., changes in attitudes as a result of deliberation) are also seen in the increase of participants viewing organoids as a research tool (increase from 22 to 33), indicating organoids primarily being a research instrument.

The discussions provided a more nuanced view of the participant perceptions of organoids; specifically, participants did not explicitly conceptualize organoids as hybrid entities, discussing them instead in the context of the person/thing dualism and, to a lesser extent, in the context of the nature/artifact dualism. Hence, in their current stage of development, a majority of participants primarily perceived organoids as research tools.

Table 3. Exemplary quotations on conceptualizations of organoids

Subject	Exemplary quotations
Conceptualizations of organoids as human	At present:<br>*I also have a concern, and as I understand it, it seems like a natural process. There is an intervention for sure. If I understood it properly, it's a natural process, the growth of cells in this way. Now I get an image—a flower that grows through the cement. That life, let's say, finds ways out* (general public, male, 41–60 years, Greece)<br>*And I think, some of the things I answered immediately are that it's something living, there's life in it. It's something living, and therefore something human. But it's also artificial, because it has been created* (religious CSO, female, 61+ years, Denmark)<br>In the future:<br>*Today, the brain organ is just a collection of cells [...] Today, research must go on [...] if, tomorrow, we were to have an organism that we can consider to be some living form ...*<br>(patient, female, 61+ years, Italy)
Conceptualizations of organoids as research tools	*I distinguish an organoid from an embryo. It is not an embryo. [...] So, organoids seem to me like just some research material. That is, I do not want to identify them; I think the questionnaire mentions them as if they are a research tool—neither an instrument nor a thing, which of course always leaves the question of how it can evolve. But, right now, and based on this data, I wanted to define them in that way* (donor, male, 41–60 years, Greece)<br>*My feeling is that it's a thing created from something human. I wouldn't consider these, at least at this tiny size, I wouldn't consider it something human. I would rather see it as a thing that can be used for research or whatever—that was made of something human* (vulnerable group, male, 18–40 years, Denmark)<br>(...) *It's difficult to think that a piece of 5mm could be considered a person*<br>(vulnerable group, male, 41–60 years, Italy)

Comparing organoids to research tools is seen as an in-between conceptualization in terms of the nature/artifact dualism. It is worth noting how only 2 of the 51 participants in the survey described organoids as a "thing," which is indicative of the complexity involved in perceiving organoids as "something more" than merely an object and something less than a person. In this regard, 19 participants indicate organoids to be living organisms (Table 3). Fourteen of those participants took part in the Danish deliberation, where a representative from the Protestant Church referred to organoids as something living. The "something more," as mentioned above, is highly complex, since even the very definition of what properties define a human—such as having the ability to sense and think, having a beating heart, and being composed of something derived/taken from the human body—is debatable. These participants were divided on seeing organoids as something human (based on the different conceptions presented above) or seeing them as research tools or "spare parts." This latter metaphor is also evoked in the Italian deliberation along with the description of "primitive entities;" participants emphasized the tiny size of current organoids to stress their incomparability with humans. Hence, the vast majority of the Italian participants perceived organoids as a research tool and agreed that organoids cannot be considered human beings. At some point, however, cerebral organoids could potentially be considered living beings, according to some of the Italian participants. A temporal aspect as to a division between current and future/potential technological applications also marks a conceptual distinction for how organoids are understood in the deliberations.

Public trust and science communication

Contrary to the Danish deliberation, the issue of public (dis)trust in science was mentioned in both the Italian and Greek workshops and displayed cross-country variation as to science and society relations as well as disparity in science and technology governance and science communication. Participants in the two deliberations associated organoid misuse concerns with a societal distrust in science. Whereas participants in the Greek deliberation related a potential misuse of data to a lack of trust in governing bodies and the intertwinement of science and political interests as reducing public trust in science, the participants in the Italian deliberation expressed general concerns for sound science communication and a particular concern as to the actual realization of the promised benefits of organoids in terms of treatment and medical advancement.

Participant recommendations for future guidelines for organoid research

A main outcome of the public deliberations includes a set of co-produced stakeholder recommendations concerning important ethical issues, challenges, and concerns to be taken into account in future guidelines for organoid

Table 4. Stakeholder priority issues for future organoid guidelines

No.	Issues
1	thorough consent procedures and resources for careful patient information
2	responsible, objective, and transparent research dissemination
3	ethical oversight and responsible governance structures
4	strict data security and storage
5	mitigation measures for unintended consequences and misuse
6	promotion of equal access to research results and therapies
7	focus on human value and improvement of the quality of life and not seeking immortality
8	revisiting and updating of guidelines and procedures in alignment with field developments

research (summarized in Table 5, 6, and 7). The intention was to elicit a broad range of perspectives and suggestions, and participants were not asked to reach consensus on a prioritized list (see detailed methods description in supplemental information for a full description of the workshop design). In the aggregate, however, *consensus* did emerge among the participants on the need to prioritize the topics included in Table 4:

Table 5, 6, and 7 summarize the co-produced main recommendations across the three workshops on the themes: “Communication and research dissemination,” “Governance of organoids,” and “Ethical implications.” The co-produced recommendations were developed on the basis of the deliberative workshop program and public engagement activities, and they mirror the key results (i.e., main issues, positions, and attitudes) identified and reported above.

DISCUSSION

To our knowledge, this is the first study conducting public deliberations on organoid research and engaging a diverse set of cross-country publics and other relevant stakeholders in informed deliberations concerning their perceptions of organoid technologies, including issues that prompt concerns, uncertainty, and/or expectations for future applications.

The study adds to the three existing patient-centered interview studies published on perspectives on the production and use of organoids (Bollinger et al., 2021; Boers et al., 2018; Haselager et al., 2020) by (1) including a diverse group of representations from the general public, patients, vulnerable groups, donors, and CSOs (sample population); (2) applying deliberative workshops as a public engagement approach to foster debate; elicit a broad range of perceptions based on expert information and Q&A sessions and co-produce a set of recommendations for future guidelines within the field (methodological approach); and (3) establishing deliberations in three different European socio-cultural contexts to increase our understanding of the potential importance of contextual factors for the formation and expression of organoid perceptions (comparative element).

Table 5. Communication and research dissemination

Topic	Main recommendations
Informed consent and patient information	(1) Informed consent procedures and forms, as well as all relevant information provided to patients and donors, should be clear, concise, simple, and understandable.<br>(2) Resources should be allocated specifically to patient/donor information (e.g., to secure sufficient time to provide information related to the donation).
Neutral, objective, and transparent research dissemination	(1) Clarity should be provided regarding how information on organoids is disclosed. It is important that information on organoids is disseminated in a clear, simple, and above all transparent manner by competent authorities to strengthen the relationship between scientific research and civil society.<br>(2) Information on the potentialities of organoid research (stage of development, progress, potential applications) must be provided to the public to allow citizens to contemplate realistic rather than unrealizable scenarios.<br>(3) Terminology is significant. Describing organoids as something human may stir fears and worries, whereas “cell cultures” and similar nomenclature come across as more neutral.<br>(4) Transparency regarding stakeholders involved in organoid research should be practiced (e.g., research performing organizations, research funding organizations, policymakers, public, and private sector).

Table 6. Governance of organoids

Topic	Main recommendations
Continuous evaluation of ethical guidelines	(1) The regular review and evaluation of ethical guidelines remains important to minimize the need to address potential issues after they become problematic. No agreement on how often this should be performed, as the progress of organoid research is unknown.
Ethical oversight	(1) The presence of ethics committees or public institutions to control how donated material and scientific discoveries are used is very important.<br>(2) Strict regulation of organoid research is needed to avoid misuse and maleficent applications, as those developed in other types of research.<br>(3) Transparency in organoid research and governance is important and will cause the public to feel safe and more trusting.
Governance responsibility	(1) Governance responsibilities regarding organoid data collection, management, and storage should be governed by the public sector.
Adaptation of guidelines in alignment with type of organoids	(1) Implementation of specific guideline updates when needed based on the particular types of organoids (e.g., cerebral organoids)
Learning and best practices implemented from guidelines on related technologies	(1) Advisable to follow the examples of other, well-established, analogous types of research/technologies and procedures (e.g., stem cell research, cloning, IVF, organ donation/transplantation, blood donation).
Data security and storage	(1) Strict focus on data security and storage is central and could be managed based on the consent given in the specific case.

Corresponding to Bollinger et al. (2021), Boers et al. (2018), and Haselager et al. (2020), we found that, across participant categories and socio-demographic differences, participants broadly supported research into and applications of organoid technologies. This is also in line with previous studies of public attitudes toward stem cell research (Allum et al., 2017; Critchley et al., 2013; Einsiedel et al., 2009; Zoeller, 2014). Here, a generally supportive attitude toward stem cell research was also identified.

In the Danish and Italian deliberations, it was observed that patients or participants who were kin to patients expressed a particularly strong sense of hope for future clinical treatments and therapies. Furthermore, in the Danish deliberation, patients acknowledged potential risks related

Table 7. Ethical implications

Topic	Main recommendations
Equal access to research results and therapies	(1) The results of biomedical research on organoids must be distributed equally and globally based on the principles of solidarity and equal access to healthcare.<br>(2) Guidelines should support a development that will not increase inequality (e.g., by avoiding monopolizing and commercialization resulting in treatments being too expensive or non-accessible to the general public.<br>(3) As organoid research and potential applications focus on the improvement of treatments, therapies, and, in general, quality of life, it should be ensured that there will be no exclusions to access due to origin, sex, sexual orientation, religious orientation, or economic status to these benefits for society at large. Organoid research should not exacerbate inequalities.
Human value and course of life	(1) Guidelines should consider current perceptions of human value and how organoid research might affect and change them.<br>(2) The extent to which intervention in the course of life is desirable is debatable. The objective must be to improve quality of life without aiming for eternal life/immortality.
Intended and unintended negative consequences	(1) The legitimate goal of saving lives, curing disease, and improving health must not affect other important ethical issues and harm others, such as the environment or other inhabitants of the Earth.<br>(2) Carefully monitoring of uses to prevent activities against humanity.<br>(3) Guidelines should include rules regarding mitigation and division of responsibility in case something goes wrong (e.g., unexpected results of organoid-based treatments).

to misuse and excessive commercialization, but some expressed that they did not worry about the use of their samples for medically relevant research. Participants in the patient group mentioned that some patients are in a position with nothing to lose concerning organoid research. This is a complex matter, and as previous research has shown, willingness to participate in clinical trials depends on a multitude of factors, including, for instance, access to healthcare, treatment options, state and nature of illness, degree and type of information provided, and societal and economic factors, among others. Common factors have been found between healthy volunteers and patients, but there are also some pertaining to each participant category (Browne et al., 2019; Moorcraft et al., 2016; Schilling et al., 2019). While more research is needed, this speaks to a potential difference in patient and non-patient perspectives as to potential differences in organoid support and risk assessments, and it equally speaks to the need for tailored, transparent, and precise information regarding the state of organoid research to avoid therapeutic misconceptions and unsupported claims concerning benefits (Baertschi et al., 2020; Bollinger et al., 2021; Bredenoord et al., 2017).

Similar to the three existing organoid studies, our findings indicate that some organoid types raise particular concerns. The case of cerebral organoids constitutes a highlighted, sensitive case due to questions concerning issues of conscience and sentience and, consequently, the moral and ontological status of organoids. It is not within the scope of this paper to review and address other types of sensitive organoids (e.g., assembloids, embryonic models, or gonadal organoids) as to their specific moral implications, but our empirical findings seem to suggest that different types of organoids warrant different challenges with respect to ethical oversight and the dissemination of science (including informed consent procedures). This study adds to the point raised by Bollinger et al. (2021, p. 1881) that it may well be insufficient to address organoid research in a uniform fashion and that additional systematic inquiries into its implications for social acceptance are required.

The lack of a broader inquiry into different types of organoids is a limitation of this study. Other potential limitations concern the representation and format of the deliberative workshop design. While broad representation has been sought across participant categories and geographical and socio-cultural backgrounds, the sample populations remain rather small, and more research is required to be able to extend the results to broader groups of patients and the public, among other stakeholders, within and across different contexts. The collective and interactional nature of the deliberative format also reduces insights into individual realities and to potential correlations between socio-demographic factors and particular formations of organoid perceptions.

The comparison of pre- and post-survey results generally indicates that the debate and expert presentations have impacted on the formations of opinions; for example, as regards the issues of organoid conceptualization and ownership. Moreover, the “don’t know” and “not answered” categories were significantly reduced (from 36 to 11 responses), which indicates an increasingly informed basis on which participants could form their own opinions.

While all participants were provided with similar information packages prior to the workshop and identical guiding questions and case material during the deliberations, the analysis indicates that variation in expert presentations influenced the emphasis on certain topics to some degree. The topic of cerebral organoids were addressed in both Danish and Italian expert presentations and were discussed to a greater length compared with the Greek debate (for an overview of expert presentations, see Table S2). Regulations and technology assessment were only addressed in the Greek expert presentations (and a reference to EU policy also only observed here) and more analogies were made to related biotechnologies, presumably due to an inclusion of this aspect in the expert presentations. While the variation in expert framing constitutes a minor limitation of this study and merely adds to the effect of a diverse set of perspectives, the centrality of a comprehensive and equally *informed* basis for inclusive deliberation (Escobar and Elstub, 2017) remains evident for this type of public dialogue.

In alignment with the existing studies referenced above, support for organoid technologies generally depends on responsible governance, ethical oversight, and sound informed consent procedures. One main concern is similarly identified as an unease toward excessive commercialization of organoids. Participants expressed concerns regarding industry monopolization and feared that organoid technologies will add to existing global healthcare inequalities if not responsibly governed. They also feared that this could increase illness hierarchies and result in over-priced therapeutics. Participants clearly emphasized the need for organoid technologies to be used in a fair, equal, and responsible manner for the public good.

Moreover, the ethical use of cell donations must be governed by strict control and responsible, transparent, and safe storage, and the use of cells, tissues, or organoids must be ensured. In this regard, participants voiced considerable concern regarding a potential misuse and breach of privacy of personal data. The deliberations revealed that issues of informed consent and proper governance structures were closely interlinked to concerns regarding insufficient data protection and commercialization issues. While this

may not be surprising, it highlights the need for wide-spanning governance structures and multi-actor efforts to safeguard the ethical and socially acceptable use of organoid technologies.

These findings also correspond with existing studies of public attitudes toward stem cell research in general. Here, similar concerns are raised about donor exploitation, commercialization, and equal access to these technologies (Einsiedel et al., 2009; Zoeller, 2014). Another shared point is that research should be publicly funded, government regulated (Allum et al., 2017; Critchley, 2008; Critchley et al., 2013), and that intellectual property rights should not be owned by a single person or company (Allum et al., 2017).

While participants took a firm stand on the commercialization issue, confirming the delicate matter of profit-making from human biological samples (Boers et al., 2018), the ownership issue raised doubts. As reflected in the pre- and post-deliberation data, 18 of 51 participants were undecided on this issue prior to the deliberations, whereas this number was reduced to only 5 participants following debate, testifying to the value of public engagement activities also in this area.

A clear majority of participants expressed that a "hospital/research institution/biobank" should own the organoids. The questions of ownership and compensation were closely interlinked in the Greek debate, indicating that the owner, being the donor or research institution, should be compensated. While this bespeaks a preferred market model for tissue exchange, overall, participant perceptions of donation are mainly aligned with the "dominant non-commercialization principle in human tissue exchange" (Boers et al., 2019, p.137), as the donation of cells is seen as being similar to blood donation, transferred without financial compensation according to the traditional gift model. The hybrid status of organoids has triggered debate in the scientific literature as to whether traditional and dichotomous gift/market models of ownership and benefit sharing are equipped to manage the pace of the growing commercialization of organoid and related stem cell technologies or whether new governance structures are needed (Boers et al., 2018). The deliberations speak to the complexity of the matter, and while participants did not reach consensus, they recommended that specifications of ownership and remuneration should be included in the consent form in agreement with the type of consent provided.

Central to the deliberations and the participant recommendations is the prioritization of transparent and objective dissemination of science, including thorough consent procedures and allocated resources for careful patient information. Participants viewed informed donor consent as a central means to safeguarding donor rights, protect autonomy and self-determination, and to be properly informed about the purpose and use of the donation. There is a potential asymmetry between requests to be fully informed and the challenges of detailing specific organoid purposes in advance of research (Baertschi et al., 2020; Mollaki, 2021). While participants agree to some restrictions being posed on the informed consent, they are divided in their preference for consent strategy (between broad, with some restrictions/tiered for certain areas, and dynamic, with re-consent procedures). Challenges with withdrawal as well as the practical implications of ongoing consent are also discussed. Public and patient perceptions of informed consent procedures could help inform policy and research discussions as to how to design and implement consent strategies and conditions suitable for organoid biobanking and clinical use, as such consensus currently does not exist (Boers and Bredenoord, 2018; Lewis and Holm, 2022; Mollaki, 2021).

Relatedly, participants also call for attention toward applied terminology in communications concerning organoid technologies. It is pointed out that descriptions of organoids as human may cause unnecessary concerns ("mini-brains" constitute one example), whereas the cell cultures concept and related designations signal greater neutrality. Similar to the studies by Bollinger et al. (2021) and Haselager et al. (2020), science fiction-like scenarios are mentioned. The example of dual use is applied to a smaller extent, and mythological references such as "Frankenstein" and "Wolverine" are mentioned in the Greek workshop. In this regard, the most explicit concern voiced is a resistance to interfering with the natural course of life and achieving immortality. The communication of scientific representations can affect public expectations and understandings and positively or negatively frame the implementation of emerging technologies and associated policy debates (Caulfield et al., 2016). In this regard, the updated "ISSCR Guidelines for Stem Cell Research and Clinical Translation" provide a set of useful recommendations for responsible conduct and scientific dissemination for researchers and research-performing institutions when communicating with the general public (International Society for Stem Cell Research, 2021).

Conclusions

Despite wide support for organoid technologies, the public deliberations carried out in this study identified a broad set of potential concerns and ethical implications for organoid research. The uncertainties raised and the recommendations suggested can help inform researchers, research-performing institutions, biobanks, research ethic committees, research integrity bodies, and patient associations in their interactions and communications with patients and publics and in their efforts to support responsible and ethical approaches within the field of organoid research. Another

key message from this study is that the sound and responsible dissemination of science not only reduces science "hype" but is also likely to reduce public uncertainties and unwarranted concerns and can help foster public acceptance and build public trust in organoid research.

EXPERIMENTAL PROCEDURES

Three deliberative workshops were conducted in November 2021 in Denmark, Greece, and Italy by the nationally positioned authors (T.R., M.P.S., and L.I.S., in Denmark; M.P., R.P., and E.C. in Italy; and E.S., V.S., and P.K. in Greece). Overall, all deliberations followed and applied the same protocol, design, and material developed by the study coordinators (T.R. and M.P.S.). The study aimed to explore and understand public perceptions and opinions on organoid research and give voice to patients, vulnerable groups, donors, and CSOs to explore a diverse set of ethical issues, scientific understandings, and societal values related to organoid technologies. The study engaged a total of 51 participants (Italy n = 19, Denmark n = 20, Greece = 12), and a purposeful maximum-variation sample strategy was applied to secure diversity in representation as a cross-section of relevant views. Nationally located authors recruited participants. The participants were all provided with a detailed information letter, including information concerning the Aarhus University privacy policy, and they all signed a written, informed consent prior to the deliberations. The Aarhus University Research Ethics Committee ethically approved the study (approval no. 2021-96). Each deliberative workshop was organized as a 1-day weekend workshop at a conference facility, professionally moderated, and structured around a two-phase deliberation process entailing two expert presentations in each workshop in addition to small- and plenum-group discussions. The deliberations were audio recorded and subsequently transcribed and coded using NVivo software. Additional information concerning the design, study method, and expert presentations is provided in the supplemental information.

SUPPLEMENTAL INFORMATION

Supplemental information can be found online at https://doi.org/10.1016/j.stemcr.2023.03.003.

AUTHOR CONTRIBUTIONS

Conceptualization, T.R. and M.P.S.; methodology, T.R. and M.P.S.; investigation, T.R., M.P.S., E.C., P.K., R.P., M.P., L.I.S., E.S., and V.S.; formal analysis, T.R., M.P.S., E.C., P.K., R.P., M.P., L.I.S., E.S., and V.S.; validation, T.R., M.P.S., E.C., P.K., R.P., M.P., E.S., and V.S.; writing – original draft, T.R.; writing – review & editing, T.R., M.P.S., E.C., P.K., R.P., M.P., E.S., and V.S.; project administration, T.R. and M.P.S.; supervision, T.R. and M.P.S.

ACKNOWLEDGMENTS

The HYBRIDA project (https://hybrida-project.eu/) has received funding from the European Union Horizon 2020 research and innovation program under grant agreement no. 101006012. The authors would further like to express our gratitude to the 51 participants in the 3 deliberative workshops. Without you generously sharing your hopes, expectations, worries, and fears regarding organoids and organoid research with us, there would have been no article.

CONFLICT OF INTERESTS

No conflicts of interest.

Received: August 19, 2022
Revised: March 1, 2023
Accepted: March 2, 2023
Published: March 30, 2023

REFERENCES

Allum, N., Allansdottir, A., Gaskell, G., Hampel, J., Jackson, J., Moldovan, A., Priest, S., Stares, S., and Stoneman, P. (2017). Religion and the public ethics of stem-cell research: attitudes in Europe, Canada and the United States. PLoS One *20*, e0176274. https://doi.org/10.1371/journal.pone.0176274.

Baertschi, B., Atlan, H., Baum-Botbol, M., Bed'Hom, B., Combrisson, H., Dosquet, C., Dubart-Kupperschmitt, A., Hirsch, F., Jouannet, P., Rémy-Jouet, I., et al. (2020). Organoids research: What are the ethical issues? Inserm-03117706, pp. 1–20.

Boers, S.N., de Winter-de Groot, K.M., Noordhoek, J., Gulmans, V., van der Ent, C.K., van Delden, J.J.M., and Bredenoord, A.L. (2018). Mini-guts in a dish: perspectives of adult Cystic Fibrosis (CF) patients and parents of young CF patients on organoid technology. J. Cyst. Fibros. *17*, 407–415. https://doi.org/10.1016/j.jcf.2018.02.004.

Boers, S.N., and Bredenoord, A.L. (2018). Consent for governance in the ethical use of organoids. Nat. Cell Biol. *20*, 642–645. https://doi.org/10.1038/s41556-018-0112-5.

Boers, S.N., van Delden, J.J.M., and Bredenoord, A.L. (2019). Organoids as hybrids: ethical implications for the exchange of human tissues. J. Med. Ethics *45*, 131–139. https://doi.org/10.1136/medethics-2018-104846.

Bollinger, J., May, E., Mathews, D., Donowitz, M., and Sugarman, J. (2021). Patients' perspectives on the derivation and use of organoids. Stem Cell Rep. *16*, 1874–1883. https://doi.org/10.1016/j.stemcr.2021.07.004.

Bredenoord, A.L., Clevers, H., and Knoblich, J.A. (2017). Human tissues in a dish: the research and ethical implications of organoid technology. Science *355*. eaaf9414–7. https://doi.org/10.1126/science.aaf9414.

Browne, J.L., Rees, C.O., van Delden, J.J.M., Agyepong, I., Grobbee, D.E., Edwin, A., Klipstein-Grobusch, K., and van der Graaf, R. (2019). The willingness to participate in biomedical research involving human beings in low- and middle-income countries: a systematic review. Trop. Med. Int. Health *24*, 264–279. https://doi.org/10.1111/tmi.13195.

Caulfield, T., Sipp, D., Murry, C.E., Daley, G.Q., and Kimmelman, J. (2016). Confronting stem cell hype. Science *352*, 776–777. https://doi.org/10.1126/science.aaf4620.

Chneiweiss, H., Dubart-Kupperschmitt, A., Baertschi, B., Galzi, J.L., Sébastianni, C., Haiech, J., and Andreescu, I. (2022). D5.1: Operational guidelines for the field of organoids and organoid-related technologies. Draft, unpublished version. HYBRIDA.

Critchley, C.R. (2008). Public opinion and trust in scientists: the role of the research context, and the perceived motivation of stem cell researchers. Public Underst. Sci. *17*, 309–327. https://doi.org/10.1177/0963662506070162.

Critchley, C.R., Bruce, G., and Farrugia, M. (2013). The impact of commercialisation on public perceptions of stem cell research: exploring differences across the use of induced pluripotent cells, human and animal embryos. Stem Cell Rev. Rep. *9*, 541–554. https://doi.org/10.1007/s12015-013-9445-4.

Einsiedel, E., Premji, S., Geransar, R., Orton, N.C., Thavaratnam, T., and Bennett, L.K. (2009). Diversity in public views toward stem cell sources and policies. Stem Cell Rev. Rep. *5*, 102–107. Epub 2009 Apr 22. PMID: 19521801. https://doi.org/10.1007/s12015-009-9063-3.

Escobar, O., and Elstub, S. (2017). Forms of mini-publics: an introduction to deliberative innovations in democratic practice. (Research and Development Notes) (newDemocracy Foundation), pp. 1–15. https://www.newdemocracy.com.au/docs/researchnotes/2017_May/nDF_RN_20170508_FormsOfMiniPublics.pdf.

Farahany, N.A., Greely, H.T., Hyman, S., Koch, C., Grady, C., Paşca, S.P., Sestan, N., Arlotta, P., Bernat, J.L., Ting, J., et al. (2018). The ethics of experimenting with human brain tissue. Nature *556*, 429–432. PMID: 29691509; PMCID: PMC6010307. https://doi.org/10.1038/d41586-018-04813-x.

Gaillard, M., Pence, C., and Botbol-Baum, M. (2021). D1.2: Identification and discussion of conceptual uncertainties relating to organoids, chimeric entities, and hybrids (HYBRIDA. EU Commission), pp. 1–26.

Haselager, D.R., Boers, S.N., Jongsma, K.R., Vinkers, C.H., Broekman, M.L., and Bredenoord, A.L. (2020). Breeding brains? Patients' and laymen's perspectives on cerebral organoids. Regen. Med. *15*, 2351–2360. https://doi.org/10.2217/rme-2020-0108.

Huch, M., Knoblich, J.A., Lutolf, M.P., and Martinez-Arias, A. (2017). The hope and the hype of organoid research. Development *144*, 938–941. https://doi.org/10.1242/dev.150201.

HYBRIDA (2023). Embedding a comprehensive ethical dimension to organoid-based research and related technologies. Project website. https://hybrida-project.eu/.

HYBRIDA Consortium (2020). Project description (European commission).

International Society for Stem Cell Research (2021). ISSCR guidelines for stem cell research and clinical translation, 2021 update. ISSCR Guidelines for Stem Cell Research and Clinical Translation, Version 1.1.

Kim, J., Koo, B.K., and Knoblich, J.A. (2020). Human organoids: model systems for human biology and medicine. Nat. Rev. Mol. Cell Biol. *21*, 571–584. https://doi.org/10.1038/s41580-020-0259-3.

Klingler, C., Wiese, L., Arnason, G., and Ranisch, R. (2022). Public engagement with brain organoid research and application: lessons from genome editing. AJOB Neurosci. *13*, 98–100. https://doi.org/10.1080/21507740.2022.2048733.

Lensink, M.A., Jongsma, K.R., Boers, S.N., Noordhoek, J.J., Beekman, J.M., and Bredenoord, A.L. (2020). Responsible use of organoids in precision medicine: the need for active participant involvement. Development *147*, 1–5. https://doi.org/10.1242/DEV.177972.

Lewis, J., and Holm, S. (2022). Organoid biobanking, autonomy and the limits of consent. Bioethics *36*, 742–756. https://doi.org/10.1111/bioe.13047.

Marsee, A., Roos, F.J., Verstegen, M.M., Roos, F., Verstegen, M., Clevers, H., Vallier, L., Takebe, T., Huch, M., Peng, W.C., and Forbes, S. (2021). Building consensus on definition and nomenclature of hepatic, pancreatic, and biliary organoids. Cell Stem Cell *28*, 816–832. https://doi.org/10.1016/j.stem.2021.04.005.

Mollaki, V. (2021). Ethical challenges in organoid use. BioTech. *10*, 12–19. https://doi.org/10.3390/biotech10030012.

Moorcraft, S.Y., Marriott, C., Peckitt, C., Cunningham, D., Chau, I., Starling, N., Watkins, D., and Rao, S. (2016). Patients' willingness to participate in clinical trials and their views on aspects of cancer research: results of a prospective patient survey. Trials *17*, 1–12. https://doi.org/10.1186/s13063-015-1105-3.

O'Brien, N., Law, S., Proulx-Boucher, K., Ménard, B., Skerritt, L., Boucoiran, I., Cox, J., Andersson, N., and de Pokomandy, A. (2020). Codesigning care improvements for women living with HIV: a patient-oriented deliberative dialogue workshop in Montréal. CMAJ Open *8*, 264–272.

Schilling, I., Behrens, H., Hugenschmidt, C., Liedtke, J., Schmiemann, G., and Gerhardus, A. (2019). Patient involvement in clinical trials: motivation and expectations differ between patients and researchers involved in a trial on urinary tract infections. Res. Involv. Engagem. *5*, 2–14. https://doi.org/10.1186/s40900-019-0145-3.

Steel, D., Bolduc, N., Jenei, K., and Burgess, M. (2020). Rethinking representation and diversity in deliberative minipublics. Journal of Deliberative Democracy *16*, 46–57. https://doi.org/10.16997/jdd.398.

The Danish Board of Technology (2014). Action Catalogue. Deliberative (Mini-publics) Workshops. Engage2020. http://actioncatalogue.eu/search.

The National Academies of Sciences, Engineering, and medicine (2021). The Emerging Field of Human Neural Organoids, Transplants and Chimeras: Science, Ethics, and Governance (The National Academic Press).

Zoeller, K. (2014). Science and the lay perspective: lay people's involvement in assessing tissue engineering. Tissue Eng. Part A *20*, 2561–2566. https://doi.org/10.1089/ten.TEA.2013.0292.

Stem Cell Reports, Volume *18*

Supplemental Information

Public perceptions and expectations: Disentangling the hope and hype of organoid research

Tine Ravn, Mads P. Sørensen, Emma Capulli, Panagiotis Kavouras, Renzo Pegoraro, Mario Picozzi, Louise I. Saugstrup, Eleni Spyrakou, and Vana Stavridi

SUPPLEMENTAL EXPERIMENTAL PROCEDURES

Design: Format and setting

The objective of conducting deliberative workshops was to explore and elicit different views on the use and derivation of organoids and organoid research to understand the public opinion (i.e., worries, concerns, fears, uncertainty, and expectations) and key ethical issues and implications related to organoids from the point of view of citizens, vulnerable groups, patients, donors, and CSOs. As a participatory method, deliberative workshops facilitate informed deliberations and a (co) production of opinions based on expert information and in-depth dialogue (for more details, please see separate research protocol, Ravn and Sørensen, 2021). The deliberative workshop was designed to explore the following research questions:

- ***How do non-professional stakeholders and the lay public perceive organoids and organoid research?***
    - What are the participants' main worries, fears, and expectations concerning organoid research?
    - How do participants conceptualize and understand organoids (i.e., persons vs. things, moral status, mythological aspects)?
    - What are the perceived current and future benefits of organoid research according to the participants?
    - Which kinds of ethical issues or research pose concern for the participants? (i.e., particular organoid types and uses; particular ethical issues such as informed consent, ownership, and commercialization)?

The three workshops were structured as a two-phase deliberation, each phase including small group and plenary discussions (please see overview, Table S1).

Table S.1. Structure and content of the deliberative workshops. Experimental procedures.

**Phases**	**Deliberative elements**	**Specifications for small-group deliberations**
**Phase 1: Attitudes and conceptualizations**	• Introduction (ethical issues)<br>• Short ice-breaker exercise<br>• Short questionnaire<br>• Presentation by experts<br>• Small-group deliberations<br><br>• Q&A session with experts<br>• Small-group deliberation<br>• Rapporteurs from each group relay a summary of the group discussion<br>• Short summation of the first part of the deliberation	Collective group preparation of three questions to experts<br><br>Deliberation based on two guiding questions regarding attitudes toward and conceptualization of organoids:<br><br>1) What do you think of organoids? What are organoids? How can we best describe them? Do you think of them as human, as a thing, or as something in-between?<br>2) Discuss the feelings organoids evoke in you. Which words come to mind when you think about organoids? Are organoids something to be feared – or do you see a hope in them? Which worries and/or positive expectations do you have regarding organoids?

**Phase 2: Ethical implications and recommendations**	• Introduction to the second part of the deliberation<br>• Individual reading of dilemmas and individual reflections<br>• Small-group deliberations<br>• Rapporteurs from each group relay a summary of the group discussion<br>• Short presentation of recommendation posters<br>• Plenum deliberation<br>• Short questionnaire<br>• Conclusion and evaluation (debriefing)	Presentations of two dilemmas related to a) Consent, ownership, and compensation and b) Cerebral organoids and the consciousness issue<br><br>A) Discussion of dilemmas based on guiding questions<br>B) Exercise. List of recommendations for important ethical issues, challenges and concerns to be taken into account in regard to organoid research<br>Guiding question:<br>1) What are the most important ethical issues that a regulatory framework for organoid research must address?

The first phase also entailed expert presentations and a Q&A session. Table S2 summarizes the focus of the expert presentations. Professional moderators and national project partners helped facilitate the small-group discussions. Subsequent to these deliberations, a rapporteur from each group relayed a summary to the following plenary discussions for collective deliberation. In the second phase, participants also provided a set of recommendations for important ethical issues, challenges, and concerns to be taken into account in future organoid research (see Table S2).

The three deliberative workshops followed the same protocol and design. All materials were developed by the study coordinators (TR, MPS) and translated and adapted if necessary by research partners locally. Participants were provided with a set of different materials prior to the workshop in order to safeguard consent, promote voluntariness, protect privacy and confidentiality, and inform participants accurately (all documents are available upon request):

- A 23-page information kit was sent out one week prior to the date of the workshop to help facilitate an informed dialogue. The kit included three short texts:
  1. What are organoids? How are they made, and how can they be used now and potentially in the future? The text broadly introduced both techniques of pluripotent stem cells (PSCs) and adult stem cells (AdSCs) for generating organoids. In general, the introduction referred to both and did not discuss detailed overlap or distinct ethical and legal implications except for a discussion on embryoids.
  2. What are some of the ethical implications of organoid research? This section included an overview of ethical issues in relation to animal experiments, different types of organoids (i.e., cerebral organoids and embryoids), biobanks, ownership and informed consent, and research integrity and public communication.
  3. How can we understand organoids conceptually? This overview introduced questions concerning the conceptual uncertainty of organoids as hybrid entities.
  The information material also included suggestions for extra material, including links to two short YouTube videos on organoid research: 1) A 3-minute introduction to organoid research by Lie and Belmonte and the New England Journal of Medicine group and a 4-minute TED-Ed animation of "What are mini-brains?" by Madeline Lancaster.
- Invitation to participate
- Information letter to participants
- Consent form
- A note on how Aarhus University processes personal data
- Detailed workshop agenda and workshop material

Table S.2. Overview of expert presentations. Experimental procedures.			
	**Italy**		
**Experts**	**Affiliation**	**Research area**	**Description of presentation**
Dr. Teresa Rinaldi	Associate professor. Department of Biology and Biotechnology "Charles Darwin" of the Sapienza University of Rome	Research and teaching within pharmacogenomics, fermentation biotechnology, and applied geosciences and bio conservation laboratory.	The presentation entitled "Organoids" introduced the topic in general terms, explaining what organoids are, by which cells, and how they are produced and the various types of organoids that exist today. The expert illustrated the possible applications of this new biotechnology and the ethical questions it raises. It was illustrated how organoids can be used as a model for the study of genetic diseases, infectious diseases, and for the study of tumors. Finally, a number of ethical issues raised by the use of organoids were pointed out, such as the moral and legal status of organoids, the use of gene editing, the creation of chimeras, marketing, and biobank storage.
Dr. Andrea Lavazza	Senior Research Fellow in Neuroethics at the Centro Universitario Internazionale di Arezzo and Adjunct Professor at the University of Pavia. He is also a lecturer at the Master of Forensic Psychopathology and Neuropsychology at the Faculty of Psychology, University of Padua.	Research in philosophy of mind and neuroethics. His main fields of interest are free will, human enhancement (including gene editing), memory-modulation, cognitive liberty, and human cerebral organoids.	The presentation entitled "Cerebral organoids and ethical questions" focused on a particular type of organoid: cerebral organoids. A number of ethical issues were outlined, some relating to organoids in general (e.g., issues relating to informed consent and ownership of the cells used) and some specifically relating to cerebral organoids (e.g., the transfer of cerebral organoids into animal models chimeric). After an overview of the various issues raised by brain organoids, the question of whether organoids can develop a form of consciousness and how this possibility affects their moral status was explored.
	**Greece**		
Dr Olga Tzortzatou-Nanopoulou	Legal counsellor and President of the Intellectual Property Committee of the Biomedical Research Foundation of the Academy of Athens (BRFAA), European Commission Expert	Specialized in GDPR/informed consent issues and IP/patents:	The presentation with the title "Information society and the current role of bioethics: Personal data and informed consent" focused on the existing regulation on biomedical research, regarding informed consent, in particular. The expert referred to the International Chapter of Principles for Sharing Data and Bio-specimen, to the GDPR regulation and crucial bioethical questions. She also presented relevant ELSI issues as well as the role of Bioethics Committees. Finally, some cases involving violations of the

			aforementioned regulations were presented.
Dr Miltos Ladikas	Senior researcher at the Institute of Technology Assessment and Systems Analysis, Karlsruhe Institute of Technology, Germany. Adviser to the European Commission, the European Research Council, the European & Developing Countries Clinical Countries Partnership, and a number of National Research organizations on social-ethical issues in science & technology developments	Specialized in Global aspects of Technology Assessment, Responsible Innovation, Ethics in Science and Technology Policy, as well as, Science Diplomacy	The presentation with the title “Technology assessment and policy making” was relevant to connecting the Technology Assessment with policy making. The expert explained the policy making procedure for a new technology and how the public opinion is introduced to this procedure. He proceeded to the description of the policy making system of some European countries and notified the importance of the public participation to the technology assessment and policy making.
	**Denmark**		
Dr. Thomas Lykke-Møller Sørensen	Associate Professor at the Department of Biological & Chemical Engineering, Aarhus University	Research in molecular biology and biotechnology, organoid technologies, and imagining biotechnology	The presentation was based on lab experiences working with organoids. During the presentation, the participants were presented with information about the development in organoid technology and how organoids are grown and used in laboratories. The expert presented different kinds of organoids (e.g., bowel, brain, and cancer) and gave different examples of how organoids are used. The presentation included pictures and videos of organoids.
Dr. Morten Dige	Associate Professor at the School of Culture and Society, Aarhus University	Research in bioethics and moral philosophy	This presentation focused on different ethical questions related to organoid research. The main focus concerned questions regarding moral status, informed consent related to the use of human tissue, and whether it is problematic to make artificial human organoids in general. The presentation included information on what moral status is, and how attitudes to who/what has moral status have changed over time. The participants were also introduced to different types of consent and how consent relates to governance in organoid research, and the relevance of commercialization, patents, and consent as issues in

			relation to the handling, distribution, and storing of stem cells.

Sampling and recruitment

Based on a separate research protocol on sampling strategy and procedures for recruitment (for details, see Ravn and Sørensen 2021a), national partners recruited participants for the deliberative workshops locally. A common excel sheet was developed to document and facilitate the recruitment process. To secure diversity in representation, a purposeful maximum-variation strategy was applied and the following participant categories were selected and included:

- The general public (e.g. variation as to age, gender, socio-economic group, ethnic background, and religious view. Members of the LGDBQ+ community was also represented)
- Vulnerable groups (e.g. including relatives to patients with genetic diseases, parents to children with genetic diseases, patients self-identifying as vulnerable)
- Patients (including e.g. patients with cystic fibrosis, ALS, gastrointestinal diseases, or cancer). Patients could also be donors.
- Donors (healthy donors donating different types of biological material)
- Civil society organizations (including e.g. religious organizations, patient organizations, science outreach organizations, blood donation organisation, students associations, consumers association)

A broader representation between participants unfamiliar with organoids and key non-professional actors (or enclave groups) representing minor groups of the public with particular experiences and/or interests in organoid research was seen as valuable to elicit a range of attitudes and explore a diverse set of organoid perceptions and understandings. In addition to ensuring variation among types of participant categories, we sought to secure diversity across age, gender, socio-economic groups, ethnic background, and religious views. The geographical coverage in terms of workshop locations was also sought to include cultural and religious variation and difference regarding the role of science in society. Table S3 provides an overview of the socio-demographic characteristics of the 51 participants participating in one of the three deliberations.

Table S3: Socio-demographic characteristics of the 51 participants. Table 1.

Socio-demographic information on participants – locally and combined	Sub-categories	Countries			Total per sub-category
		Denmark	Greece	Italy	
Distribution across participant categories	General public	4	9	3	**16**
	Vulnerable group	6	-	2	**8**
	Patient	5	1	5	**11**
	CSO	8	1	9	**18**
	Donor	3	1	1	**5**
	Not answered	-	-	-	**-**
	Total	26	12	20	**58**[1]
Age distribution	18–30	3	2	4	**9**
	31–40	2	6	4	**12**

[1] 51 people participated in the workshops. However, some participants (in Denmark and Italy) checked more than one box to categorize themselves (e.g., both 'CSO' and 'patient'). The 20 Danish participants checked 26 boxes, while the 19 Italian participants checked 20 boxes.

	41–50	3	3	3	**9**
	51–60	5	-	3	**8**
	61–70	4	1	3	**8**
	71–80	3	-	1	**4**
	81+	-	-	-	**-**
	Not answered	-	-	1	**1**
Gender distribution	Male	13	7	12	**32**
	Female	7	4	6	**17**
	Non-binary	-	1	-	**1**
	Not answered	-	-	1	**1**
Religiosity	Not religious	4	7	5	**16**
	Christian, Orthodox	1	3	-	**4**
	Christian, Catholic	1	-	10	**11**
	Christian, Protestant	12	-	-	**12**
	Christian, other	-	-	-	**-**
	Muslim	1	-	1	**2**
	Other	-	-	-	**-**
	Not answered	1	2	3	**6**
Participants' relation to job market	Employed, low income	-	1	2	**3**
	Employed, middle income	5	9	8	**22**
	Employed, high income	4	1	1	**6**
	Part-time employed	2	-	-	**2**
	Unemployed	-	1	-	**1**
	Retired	6	-	3	**9**
	Student	2	-	3	**5**
	Self-employed	1	-	-	**1**
	Not answered	-	-	2	**2**
Ethnicity/ nationality	Danish	18	-	-	**18**
	Greek	-	12	-	**12**
	Italian	-	-	16	**16**
	European (other than Danish, Greek, and Italian)	2	-	1	**3**
	Non-European	-	-	1	**1**
	Not answered	-	-	1	**1**

Participants were recruited using different strategies:

- Representatives from the public were recruited through a diverse set of media outlets, such as Facebook groups, Twitter, LinkedIn, networks, and well as through political organizations, student organizations, minority organizations etc.
- Vulnerable groups, donors and patients were recruited through patient organizations, support networks, donation organizations, flyers in outpatient clinic waiting rooms, and networks of clinicians.
- CSOs were contacted through existing networks, different associations, or directly through organizational gatekeepers.

In the recruitment and engagement of participants, attention was paid to the need for increased sensitivity to individual and contextual circumstances while adhering to the "recruitment etiquette"

building on the Belmont principles (Gyure et al. 2015, p. 2). Workshop locations were chosen to minimize travel for participants. All travel costs and potential accommodation expenses were reimbursed, and the workshops included catering and a post-workshop dinner. Participants were not given an honorarium but did receive a gift bag as a thank you for their participation.

Data Analysis

The deliberative workshops were audio-recorded for subsequent transcription, data coding, and within-case analysis. National deliberations were locally transcribed verbatim, coded, and analyzed according to a common preliminary codebook, analytical strategy, and template. The within-case analyses were then imported to the NVivo 12 software program and thematically coded (by TR) according to the main- and sub-themes conveyed, building on the pre-defined codebook deductively derived from the research questions, workshop themes, and guiding questions applied. The coding strategy also remained open to inductively derived codes emerging through the coding process to allow for new themes and across case explorations. The across-case analysis included a thematic comparison of the three deliberations, identified differences, and similarities in terms of issues raised, as well as explored contextual and conceptual variation.

Pre- and Post-Deliberation Questionnaire

As part of the deliberative workshops, the participants were asked to fill out a questionnaire in the morning, at the beginning of the workshop before the deliberations began, and again after the deliberations, at the end of the day. The questionnaire included four questions regarding the participants' understanding of and feelings toward organoids as well as questions on ownership and consent, which were asked both before and after the deliberations to measure changes in opinions as a result of the workshop deliberations (see Table S4 for results). The first part of the questionnaire also included socio-demographic questions (see results in Table S3).

Questionnaire to participants in deliberative workshops on organoids

QUESTIONS FOR THE MORNING

1. WHICH PARTICIPANT CATEGORY BEST DESCRIBES YOU?

a. VULNERABLE GROUPS (E.G., PARENTS TO CHILDREN WITH GENETIC DISEASES)
b. PATIENT (E.G., PATIENTS WITH GENETIC DISEASES SUCH AS CYSTIC FIBROSIS, CANCER, NEUROLOGIC DISEASES, GASTROINTESTINAL DISEASE, MACULAR GENERATION AMONG OTHERS)
c. DONORS (HEALTHY DONORS DONATING DIFFERENT TYPES OF BIOLOGICAL MATERIAL)
d. CIVIL SOCIETY ORGANIZATIONS, INCLUDING RELIGIOUS ORGANIZATIONS
e. GENERAL PUBLIC
f. PREFER NOT TO ANSWER THIS QUESTION

2. WHICH AGE GROUP DO YOU BELONG TO?

a. 18–30 YEARS OLD
b. 31–40 YEARS OLD
c. 41–50 YEARS OLD

d. 51–60 YEARS OLD
e. 61–70 YEARS OLD
f. 71–80 YEARS OLD
g. 81+ YEARS OLD
h. PREFER NOT TO ANSWER THIS QUESTION

3. WHICH GENDER CATEGORY BEST DESCRIBES YOU?

a. FEMALE
b. MALE
c. NON-BINARY
d. PREFER NOT TO ANSWER THIS QUESTION

4. DO YOU CONSIDER YOURSELF RELIGIOUS?

a. NO
b. YES, CHRISTIAN (ORTHODOX)
c. YES, CHRISTIAN (CATHOLIC)
d. YES, CHRISTIAN (PROTESTANT)
e. YES, CHRISTIAN (OTHER)
f. YES, MUSLIM (ALL TYPES)
g. YES, OTHER (THAN MUSLIM OR CHRISTIAN)
h. PREFER NOT TO ANSWER THIS QUESTION

5. WHICH LABOR MARKET CATEGORY BEST DESCRIBES YOU?

a. EMPLOYEE, LOW INCOME
b. EMPLOYEE, MIDDLE INCOME
c. EMPLOYEE, HIGH INCOME
d. EMPLOYEE, PART-TIME
e. UNEMPLOYED
f. RETIRED
g. STUDENT
h. PREFER NOT TO ANSWER THIS QUESTION

6. WHAT IS YOUR ETHNIC/NATIONAL BACKGROUND?

a. DANISH
b. GREEK
c. ITALIAN
d. EUROPEAN (OTHER THAN DANISH/GREEK/ITALIAN)
e. NON-EUROPEAN
f. PREFER NOT TO ANSWER THIS QUESTION

7. WHICH WORDS BEST DESCRIBE AN ORGANOID? PLEASE CHOOSE UP TO THREE ANSWERS

a. THING
b. HUMAN
c. HALF HUMAN, HALF THING
d. LIVING ORGANISM
e. SOMETHING MECHANICAL
f. ARTIFICIAL
g. NATURAL
h. CLINICAL TOOL
i. RESEARCH TOOL

j. Cell culture
k. Mini-organ
l. Science fiction
m. Frankenstein-like
n. In doubt/don't know
o. Prefer not to answer this question

8. Which of the following words (or word combinations) best describe how you feel about organoids? Please choose two words

a. Fear
b. Hope
c. Positive expectation
d. Worries
e. Progress
f. Danger
g. Excitement
h. Other feelings
i. Does not evoke any feelings
j. Prefer not to answer this question

9. Who owns the organoids?

a. The donor of the cells
b. The researcher/doctor
c. The hospital/research institution/company/biobank
d. The state
e. Don't know
f. Prefer not to answer this question

10. Which type of consent should donors give for the use of their cells for organoid research?

a. Blanket consent (i.e., material is donated without any restrictions)
b. Dynamic consent (i.e., on-going engagement and communication between donors and users)
c. Don't know
d. Prefer not to answer this question

QUESTIONS FOR THE AFTERNOON

11. Which words best describe an organoid? Please choose up to three answers

a. Thing
b. Human
c. Half human, half thing
d. Living organism
e. Something mechanical
f. Artificial
g. Natural
h. Clinical tool
i. Research tool

j. CELL CULTURE
k. MINI-ORGAN
l. SCIENCE FICTION
m. FRANKENSTEIN-LIKE
n. IN DOUBT/DON'T KNOW
o. PREFER NOT TO ANSWER THIS QUESTION

12. WHICH OF THE FOLLOWING WORDS (OR WORD COMBINATIONS) BEST DESCRIBE HOW YOU FEEL ABOUT ORGANOIDS? PLEASE CHOOSE TWO WORDS

a. FEAR
b. HOPE
c. POSITIVE EXPECTATION
d. WORRIES
e. PROGRESS
f. DANGER
g. EXCITEMENT
h. OTHER FEELINGS
i. DOES NOT EVOKE ANY FEELINGS
j. PREFER NOT TO ANSWER THIS QUESTION

13. WHO OWNS THE ORGANOIDS?

a. THE DONOR OF THE CELLS
b. THE RESEARCHER/DOCTOR
c. THE HOSPITAL/RESEARCH INSTITUTION/COMPANY/BIOBANK
d. THE STATE
e. DON'T KNOW
f. PREFER NOT TO ANSWER THIS QUESTION

14. WHICH TYPE OF CONSENT SHOULD DONORS GIVE FOR THE USE OF THEIR CELLS FOR ORGANOID RESEARCH?

a. BLANKET CONSENT (I.E., MATERIAL IS DONATED WITHOUT ANY RESTRICTIONS)
b. DYNAMIC CONSENT (I.E., ONGOING ENGAGEMENT AND COMMUNICATION BETWEEN DONORS AND USERS)
c. DON'T KNOW
d. PREFER NOT TO ANSWER THIS QUESTION

Pre- and Post-Deliberation Questionnaire Results

Table S4 displays the results of the four repeated questions in the Pre- and Post-deliberation questionnaire on participants' understanding of and feelings toward organoids, ownership, and consent.

Table S4. Pre- and post-questionnaire results. Results.

		Denmark		Greece		Italy		Combined	
		Before	After	Before	After	Before	After	Before	After
**Words describing**	**Thing**	-	1	-	-	2	1	2	2
	**Human/person**	1	1	1	-	1	-	3	1
	**Half person/half thing**	-	-	-	-	3	1	3	1

	**Living organism**	10	14	3	1	7	4	20	19
	**Something mechanical**	-	-	1	-	0	1	1	1
	**Artificial**	3	3	6	4	6	1	15	8
	**Natural**	2	3	1	2	2	2	5	7
	**Clinical tool**	1	2	2	3	2	4	5	9
	**Research tool**	7	11	3	7	12	15	22	33
	**Cell culture**	11	12	7	3	7	6	25	21
	**Mini-organ**	10	11	1	3	5	3	16	17
	**Science-fiction**	-	-	-	-	-	-	-	-
	**Frankenstein-like**	-	-	-	-	-	-	-	-
	**In doubt/don't know**	3	-	2	1	-	-	5	1
	**Not answered**	-	-	-	-	1	-	1	-
	**Total**	**48**	**58**	**27**	**24**	**48**	**38**	**123**	**120**
**Feelings related to organoids**	**Fear**	1	3	2	-	-	-	3	3
	**Hope**	8	10	5	4	12	14	25	28
	**Positive expectations**	13	13	7	10	8	9	28	32
	**Worries**	4	9	3	4	1	1	8	14
	**Progress**	9	8	3	1	9	12	21	21
	**Danger**	0	1	1	1	1	1	2	3
	**Excitement**	5	6	1	-	1	1	7	7
	**Other feelings**	1	-	-	-	-	-	1	-
	**No feelings**	-	-	1	1	-	-	1	1
	**Not answered**	-	-	-	-	2	-	2	-
	**Total**	**41**	**50**	**23**	**21**	**34**	**38**	**98**	**109**
**Who owns organoids?**	**The cell donor**	5	6	4	-	4	-	13	6
	**Researcher/doctor**	1	3	1	2	2	1	4	6
	**Hospital/research institution/biobank**	6	10	1	5	11	16	18	31
	**The state**	-	4	-	-	1	1	1	5
	**Don’t know**	11	1	5	3	2	1	18	5
	**Not answered**	-	-	1	2	2	-	3	2
**Opinions on consent**	**Without restrictions**	3	3	-	2	3	2	6	7
	**Some/certain restrictions**	2	4	4	4	4	5	10	13
	**Specified consent for certain diseases or areas**	4	2	-	1	4	5	8	8
	**Ongoing consent**	8	10	6	3	6	7	20	20
	**Don’t know**	3	-	2	-	-	-	5	-
	**Not answered**	-	1	-	2	2	-	2	3

Supplemental References

Gyure, M.E., Quillin, J.M., Rodríguez, V.M., Markowitz, M.S., Corona, R., Borzelleca, J., Jr, Bowen, D.J., Krist, A.H., and Bodurtha, J.N. (2014). Practical considerations for implementing research recruitment etiquette. *IRB*, *36*(6), 7–12.

Ravn, T., and Sørensen, M.P. (2021). D.4.1. Protocol. Organoid-based research: Engagement, co-creation and validation, HYBRIDA. EU Commission.

Ravn, T., and Sørensen, M.P. (2021a). D.4.2. Report on participant selection and procedures and criteria for recruitment. HYBRIDA. EU Commission.
